# Hearty miR-363 controls *HAND1* in cardiac cell specification

**DOI:** 10.1186/scrt478

**Published:** 2014-07-29

**Authors:** Manoj K Gupta, Tata Nageswara Rao

**Affiliations:** Section of Islet Cell and Regenerative Biology, Joslin Diabetes Center, Harvard Medical School, Boston, MA 01125 USA; Department of Stem Cell and Regenerative Biology, Harvard University, Harvard Stem Cell Institute, Cambridge, MA 01238 USA

## Abstract

MicroRNAs regulate target gene expression post-transcriptionally in a myriad of cell types and play critical roles in diverse physiological and pathological processes, including cardiomyocyte development, differentiation, and regeneration. The recent publication in *Stem Cell Research and Therapy* by Wagh and colleagues reports a novel regulatory role for miR-363 in cardiomyocyte specification. By employing microRNA expression profiling and functional knockdown studies on human embryonic stem cell-derived cardiomyocytes, the authors identified miR-363 as an upstream negative regulator of left ventricular specification transcription factor *HAND1*.

The efficient derivation of specialized and functional cardiomyocytes (CMs) from pluripotent stem cells is a primary goal for stem cell-based cardiac regenerative therapies. Such a prospect is currently hampered, however, in part by an incompletely defined complex of molecular regulators of cardiac cell development and differentiation.

This commentary discusses the findings from Wagh and colleagues published in this issue of *Stem Cell Research and Therapy* demonstrating a critical role for miR-363 in post-transcriptional regulation of CM differentiation via the hand and neural crest derivative expressed *HAND1* transcription factor [1]. Teleost fishes and amphibians show robust regeneration abilities of heart tissues, whereas the adult mammalian heart poorly renews itself, showing extremely limited regenerative capacity. CM proliferative capacity rapidly ceases shortly after birth in mammals [2]. There is little evidence for cardiac cell renewal in humans [3], indicating the limited proliferation capacity of adult CMs. During myocardium infarction or heart failure, the extensive loss of CMs leads to progressive cardiac dysfunction and chronic heart failure, a leading cause of morbidity and mortality in the developed world. Identifying molecular regulators and critical mediators of cardiac cell type development, proliferation, and differentiation is of great clinical importance, and unraveling such molecular horizons could lead to the development of therapeutic strategies for successful regeneration of the human adult heart.

MicroRNAs (miRNAs) are small noncoding RNAs (~22 nucleotides in length) that regulate gene expression post-transcriptionally by imperfect binding to the 3′ untranslated region of target mRNAs in a wide variety of cell types. Given the notion that a single miRNA may have multiple cellular targets and given the existence of vast numbers of miRNAs (~1,500 in humans), we can expect to witness the discovery of novel miRNA-dependent regulation in the modulation of versatile biological functions [4]. Recently, miRNAs have been recognized as important players in cardiac development, pathology, and regeneration. Disruption of the miRNA pathway in CMs leads to heart failure and cardiomyopathy [5]. Some miRNAs have been demonstrated to suppress CM proliferation, including miR-1, miR-133, and the miR-15 family, while other miRNAs (miR-199a and miR-590) have been shown to promote *in vivo* CM proliferation in rat and mouse models [6, 7]. miRNAs could thus be the best targets for understanding cardiac specialization during differentiation of human embryonic stem cells (hESCs). Further exploring the role of miRNAs in cardiac cells during development and disease may therefore hold great promise for cardiac therapy applications.

hESCs are a unique source for deriving cardiac cells in a specialized manner and also offer a unique opportunity to study the mechanisms and derivation of different cardiac cell types *in vitro* [8, 9]. The recent publication by Wagh and colleagues sought to identify the novel miRNAs regulating cardiac-specific transcription factors that determine left versus right ventricular determination [1]. To this end, the authors performed miRNA expression profiling in undifferentiated hESCs and CMs at day 8 and day 14 after differentiation. This screening identified 18 differentially expressed candidate miRNAs, from which they focused on miRNAs that could potentially target the 3′ untranslated regions (as predicted by *in silico* analysis) of the CM subtype specification transcription factors (*HAND1*, *HAND2*, *TBX3*, *GJA1*, *NPPA*, *RYR2*, *SLN*), and found that a subset of miRNAs (miR-363, miR-367, miR-181a and miR-181c) was expressed at a higher level in hESC-derived non-CMs compared with CMs. These novel findings were confirmed by real-time polymerase chain reaction analysis: higher expression of *HAND* genes correlated with lower expression of miR-363, miR-367, miR-181a and miR-181c. Using an elegant dual-reporter assay, the authors further showed that miR-363 specifically inhibits *HAND1* gene expression through 3′ untranslated region binding. Perhaps more heartening, overexpression of antisense oligonucleotide directed against miR-363 (anti-miR-363) during hESC differentiation promoted more *HAND1*-expressing CMs, confirming the role of miR-363 in dorso-ventral patterning and interventricular septum formation in the embryonic heart [10, 11] (Figure 1).

**Figure 1:**
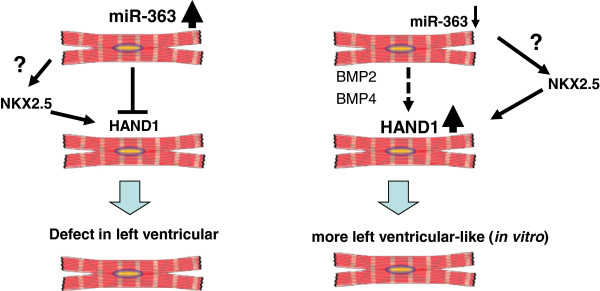
**miR-363 negatively regulates the left ventricular cardiomyocyte specification transcription factor** ***HAND1*** **.** BMP, bone morphogenetic protein; HAND, hand and neural crest derivative expressed; miR, microRNA; NKX2.5, NK2 transcription factor related, locus 5.

In summary, Wagh and colleagues demonstrated for the first time that miR-363 acts as an upstream negative regulator of *HAND1* transcription factor and extends the role of miRNAs in cardiac cell biology (Figure 1). This study has important implications for the human clinical perspective. For instance, since it is especially of great interest to derive specific cardiac subtypes from embryonic stem cell/induced pluripotent stem cell cultures and ventricular heart cells, which are the most affected cells during cardiac injury, the reversible pharmacological suppression of miR-363 may provide a novel strategy for generating functional left ventricular CMs for cardiac regenerative therapy.

Nevertheless, Wagh and colleagues’ study opens new areas of research and presents several unanswered questions for future investigations. The current study is impressive, but does the miR-363-mediated repression of HAND1 occur *in vivo*? To what extent does this regulation affect the cardiac cell specification during development? Is it safe to use anti-miR-363 for cardiac regenerative therapies? What are the additional miRNA networks that cooperate with miR-363 to form a high-order regulatory complex in determining cardiac cell specification? Most importantly, how can we derive the desired functional cardiac subtype without any genetic manipulation? These are just a few of the myriad questions that are emerging from this exciting study. Answering these questions may identify more druggable targets and might help push cardiac research much closer to human cardiac regenerative therapy.

## Abbreviations

CM: Cardiomyocyte
HAND1: Hand and neural crest derivative expressed 1
hESCs: Human embryonic stem cells
miRNA/miR: microRNA.
